# Parathyroid hormone PTH(1–34) increases the volume, mineral content, and mechanical properties of regenerated mineralizing tissue after distraction osteogenesis in rabbits

**DOI:** 10.3109/17453670903350032

**Published:** 2009-12-04

**Authors:** Ramune Aleksyniene, Jesper Skovhus Thomsen, Henrik Eckardt, Kristian G Bundgaard, Martin Lind, Ivan Hvid

**Affiliations:** ^1^Orthopaedic Division of Northern Denmark, Aalborg University Hospital, University of Aarhus, Aalborg, Denmark; ^2^Department of Connective Tissue Biology, Institute of Anatomy, University of Aarhus, Århus, Denmark; ^3^Trauma Center Murnau, Murnau, Germany; ^4^Sector for Limb Reconstruction and Paediatric Orthopaedics, Aalborg University Hospital, University of Aarhus, Aalborg, Denmark; ^5^Department of Orthopaedics, Sector for Sports Medicine, Aarhus University Hospital, Århus, Denmark; ^6^Sector for Paediatric Orthopaedics, Department of Orthopaedics, Aarhus University Hospital, Århus, Denmark

## Abstract

**Background and purpose** Parathyroid hormone (PTH) has attracted considerable interest as a bone anabolic agent. Recently, it has been suggested that PTH can also enhance bone repair after fracture and distraction osteogenesis. We analyzed bone density and strength of the newly regenerated mineralized tissue after intermittent treatment with PTH in rabbits, which undergo Haversian bone remodeling similar to that in humans.

**Methods** 72 New Zealand White rabbits underwent tibial mid-diaphyseal osteotomy and the callus was distracted 1 mm/day for 10 days. The rabbits were divided into 3 groups, which received injections of PTH 25 µg/kg/day for 30 days, saline for 10 days and PTH 25 µg/kg/day for 20 days, or saline for 30 days. At the end of the study, the rabbits were killed and the bone density was evaluated with DEXA. The mechanical bone strength was determined by use of a 3-point bending test.

**Results** In the 2 PTH-treated groups the regenerate callus ultimate load was 33% and 30% higher, absorbed energy was 100% and 65% higher, BMC was 61% and 60% higher, and callus tissue volume was 179% and 197% higher than for the control group.

**Interpretation** We found that treatment with PTH during distraction osteogenesis resulted in substantially higher mineralized tissue volume, mineral content, and bending strength. This suggests that treatment with PTH may benefit new bone formation during distraction osteogenesis and could form a basis for clinical application of this therapy in humans.

## Introduction

Parathyroid hormone (PTH) is a major regulator of bone metabolism. It is a multifunctional molecule, with a unique ability to affect the bone metabolism in either a catabolic or an anabolic way. Intermittently administered human PTH is a strong bone anabolic treatment regimen (Rubin et al. 2002, Hamann and Lane 2006). Recombinant human parathyroid hormone (rhPTH) and recombinant bioactive fragments of human PTH are emerging as a unique new class of treatment options for osteoporosis (Hamann and Lane 2006). At present, PTH is the most potent anabolic (as opposed to antiresorptive) agent available for clinical treatment of osteoporosis to reduce fracture risk (Hamann and Lane 2006).

Recent experiments with rats have demonstrated that treatment with PTH increases the mechanical strength and the callus formation in normal healing fractures (Holzer et al. 1999, Andreassen et al. 1999, 2001, Alkhiary et al. 2005, Barnes et al. 2008). Furthermore, an increased density of regenerated bone and enhanced fixation of steel implants in rats have been shown after PTH treatment (Skripitz et al. 2000b, Skripitz and Aspenberg 2001). Our knowledge of the effects of intermittent PTH treatment on newly regenerating bone after distraction osteogenesis is very limited. Seebach et al. (2004) reported enhanced mechanical strength and density of new bone after distraction osteogenesis in rats. However, no information is available on the effects of intermittent PTH treatment on distraction osteogenesis in a larger animal.

The process of new bone formation is highly coordinated through different factors, such as blood supply, cytokines, growth factors, stem cell availability, and physical stresses and strains. The tissue within the gap between distracted bone ends is pluripotent but all the factors that influence the differentiation of the cells have not been fully determined. These undifferentiated cells in the bone gap may be targets for mechanical or biological intervention designed to promote bone regeneration (Richards et al. 1999). PTH may be one of these biological factors, although the mechanism behind the anabolic effect of intermittent PTH treatment is not understood. PTH enhances the activity and number of osteoblasts, which form bone and produce and secret insulin-like growth factor I (IGF-I), which, together with other growth factors such as IGF-II and TGF-β-1, mediates bone growth and development (Watson et al. 1995). PTH changes the levels of expression of many hormones, cytokines, and growth factors (Rubin et al. 2002). Consequently, as osteoblasts are the principal cellular target of PTH, intermittent administration of PTH may be useful as a pharmacological stimulator of new bone formation during distraction osteogenesis.

Clinically, distraction osteogenesis is widely used for leg lengthening, for treatment of non-union fractures, to replace bone defects after tumor resections and infections, and for treatment of various structural deformities. In humans, the completed process of new bone formation and consolidation to a level where it has sufficient strength for weight bearing requires a period of months to years. Thus, the potential of PTH to enhance bone formation and thereby bone strength after distraction osteogenesis would be of benefit.

Our hypothesis was that intermittently administered human PTH(1–34) would enhance bone mineral content, mechanical strength, and regenerated tissue volume after standardized distraction osteogenesis in a rabbit model.

## Materials and methods

### Animals

72 skeletally mature female New Zealand White rabbits, 6–8 months old with a mean body weight of 3.96 (3.4–4.6) kg were used in the study. The animals were housed separately in standard cages in a temperature-controlled room (21 ± 2°C), with free access to food and water. General anesthesia was induced and maintained by administering 5 mL subcutaneously 20 min before the operation and 2 mL subcutaneously at the start of the operation of a mixture prepared from 20 mL Ketamine (50 mg/mL), 2.5 ml Lidocaine (20 mg/mL), 1 mL Acepromazin (10 mg/mL), and NaCl. The rabbits received postoperative pain management, which followed the approved protocol. Antibiotics were not used. The experiment was approved by the Danish Committee on Animal Experimentation (no. 2002/561-501).

After the operation, the rabbits were randomly divided into 3 groups. The animals in the first group (PTH) received a subcutaneous injection of 25 µg/kg PTH (human PTH(1–34) (Bachem, Bubendorf, Switzerland) dissolved in 0.5 M saline with 2% heat inactivated rabbit serum) on a daily basis during the lengthening and consolidation period (30 days). The animals in the second group (vehicle + PTH) were injected on a daily basis with vehicle during the lengthening period (10 days) and with PTH (25 µg/kg/day) during the consolidation period (20 days). The animals in the third group received a corresponding injection of vehicle, and served as controls. The PTH dosage was selected based on the findings of a pilot study (Aleksyniene et al. 2006).

### Surgical procedure and distraction protocol

**Figure 1.**
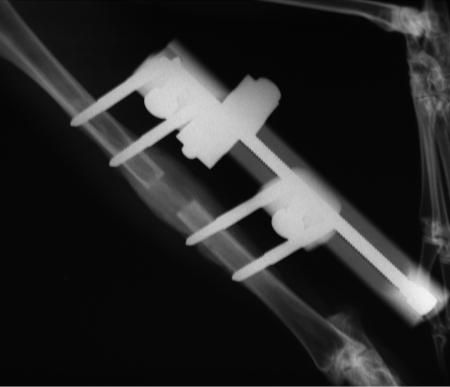
Radiograph taken during the consolidation period on day 28 (PTH group) showing both periosteal and endosteal regenerate callus.

The standard surgical approach and a distraction protocol introduced by Schumacher et al. (1994) and later modified by Bundgaard (1999) was used. The right tibia was approached through a curved longitudinal skin incision. The fascia was cut, the muscles separated, and the anterior medial surface of the tibia was opened. The periosteum was incised longitudinally and retracted. 4 holes were drilled with a 2-mm drill and self-taping screws (M300 or M301; Orthofix, Bussolenga, Italy) were inserted. The periosteum was protected and an osteotomy performed at the mid-diaphysis just below the tibiofibular junction between the second and third screws. A monolateral external fixator (Orthofix M100) was mounted. The periosteum and muscular fascia were then closed over the bone with interrupted sutures, and sutures were used to close the skin. Buprenorphinum (Temgesic) was given routinely as subcutaneous injections twice a day in order to control postoperative and lengthening-induced pain until 15 days after surgery, at a dose of 0.04 mg/kg after which the dose was gradually reduced to 0 over the following 3 days. The wound was observed daily for signs of infection. The animals were weight-bearing and mobile on the day of surgery. The distraction was started 5 days postoperatively at a rate of 1 mm/day (0.5 mm in 2 increments with at least 6 h between the increments) and continued for 10 days. Then a consolidation period of 20 days followed. Radiographs were taken under sedation 5, 14, and 28 days postoperatively in order to monitor bone formation and the fixation of the distraction device (Figure 1).

All animals were killed 5 weeks postoperatively with a barbiturate overdose given intravenously. The tibiae of both legs were disarticulated, dissected free, cleaned, radiographed in 2 planes, and frozen at –20°C.

### Dual-energy X-ray absorptiometry (DEXA)

The right and left tibial bone specimens were scanned in a DEXA scanner (Norland Eclipse; Norland, Ft. Atkinson, WI) in a standard position with the posterior surface of the bone facing the scanner plate. The bone mineral content (BMC) and areal bone mineral density (BMD) were determined with 2 regions of interest (ROIs) using the Small Subject program.

Firstly, a 15-mm-long and 30-mm-wide “total ROI” was placed centrally between the second and third screw holes (marked with injection needles), which included approximately 2.5 mm of the proximal and approximately 2.5 mm of the distal old bone and the surrounding callus. Secondly, a 6-mm-long and 30-mm-wide “de novo ROI” was placed centrally in the regenerated callus. In addition, the BMD was measured at the mid-diaphysis of the intact contralateral tibia with the same size and approximate location as the “total ROI” of the distracted tibia.

### Preparation of bone specimens

**Figure 2.**
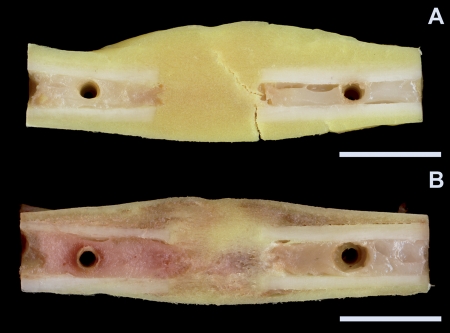
Longitudinal sections of the bone specimens made after the mechanical testing procedure: A. PTH; B. control. Bar = 10 mm.

Subsequently, an approximately 40-mm-long bone specimen containing the regenerated callus was obtained from the distracted tibia by sawing between the first and second screw holes and between the third and fourth screw holes with a diamond precision-parallel saw (Exakt Apparatebau GmbH, Norderstedt, Germany) (Figure 2). In addition, a similar specimen was obtained from the contralateral tibia.

### Bone volume and external callus dimensions

The volume of the bone specimens was determined using Archimedes' principle by weighing the specimens before and during immersion in water on an electronic scale equipped to measure volumes. The total regenerate tissue volume was estimated as the volume of the distracted 40-mm-long bone specimen minus the volume of the contralateral intact 40-mm-long bone specimen. The length of the tibial bone specimen and the distance between the second and third screw holes were determined with an electronic sliding calliper, and the midpoint of the bone specimen was marked with a waterproof marking pen. The distance between the second and third screw holes was considered to be an expression of the length gained during the distraction. In addition, the external anterior-posterior and medial-lateral diameters were measured with the sliding calliper at the midpoint of the distraction regenerate callus.

Before the destructive mechanical test, all bone specimens from the distracted tibiae were µCT scanned in order to investigate the effects of the PTH treatment on the microstructure of the regenerated mineralized tissue. The results of this investigation will be presented elsewhere.

### Mechanics

The mechanical strength of the regenerated bone was determined by a 3-point bending test (Turner and Burr 1993) in a materials-testing machine (Instron 5566; Instron, High Wycombe, UK). The bone specimens were placed with the posterior surface facing downwards on a custom-made testing jig constructed for 3-point bending. The distance between the supporting rods was fixed at 30 mm. Both distracted and intact tibial bone samples were tested at a constant deformation rate of 2 mm/min with the load applied at the midpoint of the bone specimen. During the 3-point bending test, load-deformation values were recorded using an attached PC and the software supplied with the materials testing machine. The load-deformation data were subsequently analyzed using a custom-made computer program (Mosekilde et al. 1997). The following parameters were calculated: ultimate load, F_max_ (i.e. the maximum force that the specimens sustain), stiffness (the slope of the linear portion of the load-deformation curve), and work to failure (W_abs_; the area under the load-deformation curve until failure). These measures are structural properties that depend on intrinsic material properties and on the geometry of the samples. The cross sections could not be assumed to be elliptical and homogenous, and a simple estimation of the axial moment of inertia could thus not be performed. Consequently, the data were not normalized in order to estimate geometry-independent material properties.

For the intact tibia, only the ultimate load is reported here whereas the other mechanical properties will be reported elsewhere.

### Statistics

The statistical analysis was performed using Stata (Stata Corporation, College Station, TX). Differences between groups were tested by analysis of variance (ANOVA) with a t-test for post-hoc analysis. Before the analysis, all data were tested for normal distribution and homogeneity of variances, and when these conditions were not fulfilled, the analysis was performed on logarithmically transformed data or using a Kruskal-Wallis test followed by a Wilcoxon-Mann-Whitney test for post-hoc analysis. As the final body weights differed statistically significantly between the 2 PTH-treated groups and the control group, the analysis of mechanical test data was performed using bone strength parameters adjusted to the final body weight of the animal. Unless otherwise stated, the results are presented as means and 95% confidence intervals (95% CIs). The statistical method used is indicated in the tables. Differences were considered significant at p < 0.05.

## Results

21 rabbits were excluded from the study: 18 had a tibial fracture through one of the fixation screw holes, 2 died due to repeated anesthesia during radiographic examination, and 1 died from unknown causes. In all, 51 of the 72 rabbits completed the study and were included in the analyses, with the following group distribution: PTH, 19 animals; vehicle + PTH, 17 animals; and control, 15 animals. During the experiment, there were no signs of infection except for a few mild delays in wound healing due to self-extraction of the operation suture, requiring no treatment. All rabbits lost weight during the study, on average 365 (SD 199) g. The animals that received PTH lost 10% of their original weight, animals that received vehicle + PTH lost 11%, and the control animals lost 7% (Table 1).

**Table:** Table 1. Animal body weight characteristics

	Mean (SD) [range]			p-value			
Body weight (g)	PTH (n = 19)	Vehicle + PTH (n = 17)	Control (n = 15)	ANOVA	PTH vs. Control	Vehicle+PTH vs. Control	PTH vs. Vehicle+PTH
Initial	3.94 (28) [3,500–4,450]	3,900 (304) [3,400–4,500]	4,063 (275) [3,500–4,600]	0.3	0.2	0.1	0.7
Final	3.55 (26) [3,150–3,950]	3,485 (295) [2,900–3,950]	3,787 (308) [3,200–4,300]	0.01	0.02	0.005	0.5

Radiographic follow-up provided a visual evaluation and monitoring of the regenerate tissue formation. Initiation of both periosteal and endosteal calluses at the bone ends became apparent at the end of the lengthening period, i.e. 2 weeks postoperatively (Oxlund et al. 1993). After having performed a series of radiographs, we were able to observe callus progression on both sides towards the interfragmentary gap with the characteristic zone structure, i.e. a central radiolucent zone and two adjacent sclerotic zones (Yasui et al. 1993, Li et al. 2006). Moreover, in both PTH-treated groups the regenerate callus was visually denser and larger than in the control group.

The length gained during distraction was similar for all 3 groups. The average of the indirect measurement of the length gained (the length between the second and third screw holes) was 28 (SD 0.6) mm in the PTH group, 28 (SD 0.86) mm in the vehicle + PTH group, and 27 (SD 0.84) mm in the control group. In all the animals, the tibia was lengthened by approximately 10 mm (approximately 10% of the initial tibial length).

### Regenerate tissue volume and external callus dimensions (Table 2)

The animals in the 2 PTH-treated groups (PTH and vehicle + PTH) had statistically significantly higher regenerate tissue volume and volume of the mid-shaft fragment, including regenerated callus and surrounding bone, than the control animals. The external regenerate callus dimensions were also statistically significantly higher in both PTH-treated groups than in the control group. However, for these parameters the differences between the two PTH-treated groups were not statistically significant.

**Table:** Table 2. Total regenerate callus volume and external callus dimension properties of the regenerated callus

	Mean (SD)			p-value			
	PTH (n = 19)	Vehicle + PTH (n = 17)	Control (n = 15)	**^a^** ANOVA **^b^** Kruskal-Wallis	PTH vs. Control	Vehicle+PTH vs. Control	PTH vs. vehicle+PTH
Volume (mm^3^)							
Mid-diaphyseal							
sample (40 mm)	4,030 (220)	4,129 (296)	2,536 (73)	< 0.001 ^**b**^	< 0.001	< 0.001	0.8
sample left (40 mm)	1,621 (27)	1,653 (37)	1,673 (31)	0.5 **^a^**	0.3	0.7	0.5
Regenerated callus	2,408 (211)	2,557 (299)	862 (56)	< 0.001 ^**b**^	< 0.001	< 0.001	0.9
Callus dimensions (mm)							
Anterior-posterior (AP)	13.2 (0.5)	12.7 (0.6)	10.4 (0.3)	< 0.001 **^a^**	< 0.001	0.002	0.4
Medial-lateral (ML)	13.4 (0.3)	13.3 (0.3)	11.1 (0.2)	< 0.001 **^a^**	< 0.001	< 0.001	0.9

### Dual-energy X-ray absorptiometry (Table 3)

The PTH-treated animals had statistically significantly higher BMC and BMD of the regenerated tissue in both the “total ROI” and the “de novo ROI” than the control animals. The differences between PTH-treated and vehicle + PTH-treated animals were not statistically significant. At the contralateral intact tibia, the animals in the PTH group had statistically significantly higher BMD than the control animals. In contrast, the animals in the vehicle + PTH group did not have statistically significantly higher BMD than the control animals.

**Table:** Table 3. Bone mineral content (BMC) and bone mineral density (BMD) of the regenerated callus, and BMD of the contralateral intact tibia

	Mean (SD)			p-value			
	PTH (n = 19)	Vehicle + PTH (n = 17)	Control (n = 15)	**^a^** ANOVA **^b^** Kruskal-Wallis	PTH vs. Control	Vehicle+PTH vs. Control	PTH vs. Vehicle+PTH
Total ROI							
BMC (g)	0.91 (0.12)	0.9 (0.21)	0.59 (0.84)	< 0.001 ^**a**^	< 0.001	< 0.001	0.7
BMD (mg/cm^2^)	400 (40)	390 (70)	311 (40)	< 0.001 ^**b**^	< 0.001	< 0.001	0.6
De novo ROI							
BMC (g)	0.33 (0.05)	0.33 (0.09)	0.21 (0.04)	< 0.001 ^**a**^	< 0.001	< 0.001	0.7
BMD (mg/cm^2^)	370 (40)	360 (80)	270 (40)	< 0.001 ^**b**^	< 0.001	< 0.001	0.6
Contralateral central ROI							
BMD (mg/cm^2^)	314 (20)	300 (20)	290 (20)	0.01 ^**a**^	0.004	0.3	0.05

### Mechanics (Table 4 and Figure 3)

**Figure 3.**
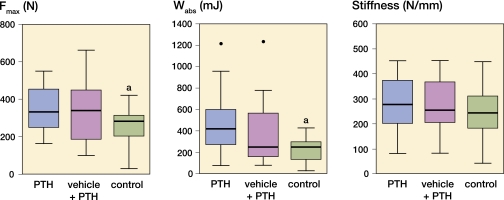
Box-and-whisker plots showing the results of the three-point bending test of the distracted tibia.

**Table:** Table 4. Results of the three-point bending test for the distracted tibia and the contralateral intact tibia. The analysis was performed using data adjusted to the final body weight of the animal (see the text for detail). Selected data are shown in Figure 3

	Mean (SD)			p-value, (95% CI for differences)			
	PTH (n = 19)	Vehicle + PTH (n = 17)	Control (n = 15)	ANOVA	PTH vs. Control	Vehicle + PTH vs. Control	PTH vs. Vehicle + PTH
Distracted tibia							
F_max_ (N)	344 (29)	338 (40)	260 (27)	< 0.001	0.001 (-228 to -60)	0.001 (66 to 242)	0.9 (-67 to 88)
W_abs_ (mJ)	454 (112)	374 (112)	227 (101)	0.02	0.01 (-489 to -100)	0.02 (31 to 437)	0.5 (-240 – 119)
Stiffness (N/mm)	283 (71)	269 (80)	250 (34)	0.2	0.3 (-141 to 19)	0.2 (-31 to 133)	0.8 (-83 to 63)
Intact contralateral tibia							
F_max_ (N)	491 (14)	453 (14)	449 (14)	0.005	0.004 (-100 to -20)	0.07 (-15 to 69)	0.2 (-70 to 3)

The ultimate load of the regenerate mineralizing callus in the PTH-treated groups was 33% higher (PTH) and 30% higher (vehicle + PTH) than that of the control group (p = 0.001 in both cases). Similarly, the work to failure in the 2 PTH-treated groups was 100% higher (PTH) and 65% higher (vehicle + PTH) than that of the control group (p = 0.01 and p = 0.02, respectively). In contrast, the ultimate load and the work to failure were not statistically significantly different between the 2 PTH-treated groups. Furthermore, the stiffness of the mineralizing callus of the PTH-treated animals was 13% higher (PTH) and 8% higher (vehicle + PTH) than that of the control group, but these differences were not statistically significant. The ultimate load of the intact tibia was 10% higher in the PTH group than in the control group (p = 0.004) , whereas the ultimate load of the animals in the vehicle + PTH group was not statistically significantly different from that of the control group.

**^a^** different from both PTH groups (p < 0.05)

## Discussion

We found that intermittently administered PTH augmented new mineralizing callus formation and development, resulting in increased regenerate callus size, volume, and bone mineral content. Moreover, the increased amount of new mineralized tissue resulted in an increase in ultimate load and work to failure of the regenerate callus in both PTH-treated groups compared to the control group.

To the best of our knowledge, only one experimental study has been conducted previously to investigate the effects of PTH on distraction osteogenesis (Seebach et al. 2004). That study was performed in rats, which have limited intracortical remodeling; it was therefore of interest to investigate the effects of PTH on distraction osteogenesis using a larger animal model with a cortical bone remodeling pattern more similar to that of human cortical bone. Rabbits have a short remodeling period compared to larger animals, they grow rapidly, and achieve early skeletal maturation (by 6–9 months) (Hirano et al. 1999). Previously, the effect of PTH on rabbits has only been investigated using intact bone (Hirano et al. 1999, Mashiba et al. 2001), whereas no previous studies have evaluated the effects of PTH on distraction osteogenesis using a larger animal such as the rabbit.

The study by Seebach et al. (2004) indicated that after 20 days of consolidation the ultimate load and the stiffness of the distracted bone were 50% higher in PTH-treated rats than in control rats. Furthermore, they showed that the total regenerate callus volume was 58% higher and the BMC and BMD of the distracted callus were 24% higher in PTH-treated rats than in control rats. Our findings in rabbits are consistent with their findings in rats, although we found that the regenerate callus BMC was approximately 60% higher in PTH-treated animals than in control animals. The effect of the PTH treatment on the mechanical properties was less pronounced than in the rat study by Seebach et al. (2004). However, it is important to note that the rat exhibits a stronger bone-anabolic response to PTH than humans do (Li et al. 1999). As rabbits have a cortical bone remodeling pattern more similar to that of human bone than to that of rats, the anabolic response to PTH in rabbits would not be expected to be as prominent as in rats. In addition, our findings confirm the results of other studies showing substantial anabolic effects on newly forming bone in different situations such as fracture healing, bone chamber studies, or titanium implant anchorage in low-density trabecular bone (Andreassen et al. 1999, Holzer et al. 1999, Skripitz et al. 2000a, Skripitz and Aspenberg 2001, Gabet et al. 2006).

In the present study, we have analyzed mechanical strength parameters that represent extrinsic properties of bone such as ultimate load, energy absorption, and bone stiffness (Turner and Burr 1993). It was not possible to normalize the size-dependent mechanical properties to geometry-independent mechanical properties, due to the irregular shape of the callus. However, the ultimate load and energy absorption are the most important measures in bone mechanics and provide good estimates of clinically relevant mechanical properties (Eckardt 2003). In PTH-treated animals, the ultimate load of the distracted tibia was 70–75% of the ultimate load of the intact contralateral tibia, whereas the ultimate load of the distracted tibia in control animals was only 58% of the ultimate load of the contralateral intact tibia.

The anabolic effect of PTH could also be detected in the intact contralateral tibia, but to a lesser extent than was observed in the distracted tibia. This supports the notion that PTH has a stronger effect on bone with an activated repair response than on bone undergoing normal remodeling (Seebach et al. 2004, Skripitz et al. 2000b).

The DEXA data were investigated with 2 different ROIs in order to analyze the entire callus including old cortical bone (“total ROI”) and the newly regenerated mineralizing tissue (“de novo ROI”) separately. The BMC of the “total ROI” of the PTH-treated animals was almost 75% of that of the contralateral intact tibia, whereas the BMC of the “total ROI” of the untreated control animals was only 15% of that of the contralateral intact tibia. This clearly demonstrates that development of new calcified tissue at the distracted tibia is accelerated in PTH-treated animals.

We studied 2 different PTH treatment regimens in order to determine whether the effect of PTH treatment during lengthening and consolidation differs from the effect of PTH treatment during the consolidation period only. However, the bone parameters analyzed were similar for the 2 PTH treatment regimens. This is in accordance with the findings of Richards et al. (1999), who showed that the bone formation in the distraction gap in rabbit bone was most active between 18 and 24 days after the operation. Furthermore, they found an increase in mineralized new bone volume and an increase in BV/TV within the distraction gap, and observed regions of both intramembranous and endochondral bone formation early in the consolidation period. Consequently, Richards et al. (1999) suggested that interventions designed to enhance the bone formation process should occur during this time window.

In addition, PTH may stimulate matrix synthesis and mineralization at early stages of the consolidation period, whereas the contribution of PTH to the very young immature pluripotent tissue during the lengthening period remains questionable. It is known that PTH also stimulates progenitor cell proliferation and recruitment of bone lining cells (Thomas 2006), although our study does not demonstrate that treatment with PTH during both the lengthening and consolidation periods is superior to treatment with PTH during the consolidation period only. In contrast, in mature bone the anabolic effect of PTH is primarily determined by bone cells that are known to be direct targets of PTH, which line the trabecular surface (Dobning et al. 1995)—and their (re)activation (Friedl et al. 2007). Osteblasts are known to be direct targets of PTH (Thomas 2006). Thus, the anabolic effect of PTH on new mineralized tissue in the later stages of the consolidation period would be expected to be more pronounced than in the earlier stages. However, our results do not demonstrate any additional benefit from treatment with PTH during the distraction period, and therefore suggest that the stimulation of bone formation with PTH could be limited to the consolidation period. Further studies are needed in order to investigate whether PTH treatment is needed for the entire duration of the consolidation period, or whether it could be limited to a shorter period. In humans, the consolidation period after distraction osteogenesis is time consuming since the maturation and consolidation of the regenerate callus typically requires a period that is 2–3 times as long as the distraction period. Identification of the part of the consolidation period that would benefit most from the PTH treatment would be cost effective.

The mechanical properties of osseous tissue depend on a number of features, which may influence the test results (Paavolainen 1978). If the mechanical equipment used for measurements is as precise as possible, biological variations may critically affect the results. Paavolainen (1978) showed that the rabbit body weight was correlated to the tibial maximum torque moment and energy absorption in a study on the healing of experimental fractures. Paavolainen (1978) therefore suggested that if the body weight of the animals differed, the torsional properties of whole bone specimens should be corrected for the weights of the animals, in order to arrive at comparable results. In our study, we observed that the animals lost approximately 9% of their initial body weight. At the end of the experiment, the body weight of the rabbits in both PTH-treated groups was lower than in the control group. Although our mechanical testing method was different from that of Paavolainen (1978), the parameters obtained in our study reflect similar bone strength characteristics and therefore the statistical analysis of mechanical strength results was performed on data adjusted to the final body weight of the rabbits.

Our findings indicate that intermittent human PTH treatment could be used for bone stimulation in orthopedic surgery, and that a positive anabolic effect of PTH on regenerate bone can be expected in humans.
